# COVID-19 non-pharmaceutical interventions: data annotation for rapidly changing local policy information

**DOI:** 10.1038/s41597-023-01979-6

**Published:** 2023-03-09

**Authors:** Benjamin Hurt, Oishee Bintey Hoque, Finn Mokrzycki, Anjali Mathew, Maryann Xue, Luka Gabitsinashvili, Haile Mokrzycki, Ranya Fischer, Nicholas Telesca, Lauren Aurelia Xue, Jacob Ritchie, J. D. Zamfirescu-Pereira, Michael Bernstein, Mark Whiting, Madhav Marathe

**Affiliations:** 1 Network Systems Science and Advanced Computing, Biocomplexity Institute and Initiative, University of Virginia, Charlottesville, USA; 2 Dept of Computer Science, Stanford University, Stanford, USA; 3 Dept of Electrical Engineering and Computer Science, University of California, Berkeley, USA; 4 Department of Computer and Information Sciences, University of Pennsylvania, Philadelphia, USA

**Keywords:** Databases, Research data, Health care

## Abstract

Understanding the scope, prevalence, and impact of the COVID-19 pandemic response will be a rich ground for research for many years. Key to the response to COVID-19 was the non-pharmaceutical intervention (NPI) measures, such as mask mandates or stay-in-place orders. For future pandemic preparedness, it is critical to understand the impact and scope of these interventions. Given the ongoing nature of the pandemic, existing NPI studies covering only the initial portion provide only a narrow view of the impact of NPI measures. This paper describes a dataset of NPI measures taken by counties in the U.S. state of Virginia that include measures taken over the first two years of the pandemic beginning in March 2020. This data enables analyses of NPI measures over a long time period that can produce impact analyses on both the individual NPI effectiveness in slowing the pandemic spread, and the impact of various NPI measures on the behavior and conditions of the different counties and state.

## Background & Summary

The spread of COVID-19 presented a significant threat to the health and safety of populations globally. In response, administrations were forced to decide on courses of action aimed at limiting that spread. Especially during the period before vaccines were broadly available, Non-Pharmaceutical Intervention (NPI) measures, such as mask mandates or stay-in-place orders, were popular. For future pandemic response, it is critical to understand the impact of these actions to ensure the most effective measures (considering both their cost and impact) are chosen. In order to reach these conclusions, the datasets of the actions taken must exist. Unfortunately, this important information has not been centrally stored and is difficult to acquire.

The design of this dataset, efficient and easy to understand, can be applied on a broad scale - reducing social cost of similar research and promoting confidence in the outcomes of such research^1^. In addition, creating this dataset, and making it widely available, contributes to the international community’s development of pandemic response strategies and policy decisions that are empirically tuned. Even using just US-based data should allow countries (or local governments) with similar features but lacking observational data to benefit^2^.

Studies on collecting Non-Pharmaceutical Intervention (NPI) measure data and how they impacted population behavior grew in number during the COVID-19 pandemic^3^. That literature focused on modeling^4,5^ and publicly available datasets^6,7^. Researchers also collected data focusing on the first and second waves at larger scale (e.g. country level)^7–14^. Some of these resulting databases contain over 60 different types of interventions, but others focused on single measures such as: lockdowns^4,15,16^, business closings^17–19^, school and college closings^17,20^, religious closings^21^, and mask mandates^22–24^.

To build the above datasets, and others, there have been several methods used to gather and validate NPI data. Researchers in the U.S.^21^ designed surveys with different questionnaires to gather U.S. county-level data, but this risked increased mislabeling since the untrained volunteers occasionally reported statewide mandates as county-level ones. Some^7,25^ collected data from different public or official resources with the help of crowdsourcing. Some researchers^9^ collaborated with government officials, while others^6,8,11,13,14^ trained contributors to collect the data and follow their individual data collection process. Still others^12^, with the help of RA’s and integrating automated processes collected data from different public resources. Some of the efforts used news articles and official press releases and briefings as evidence^13^. Table 1 summarizes some of the available NPI datasets and ours. From the table, we see that the datasets are collected for a limited time frame (e.g. based on first/second wave) across varying geographical dimensions.

**Table 1:** Some of the available NPI datasets. Our data spans over two years of the ongoing pandemic and covers an entire state, making it the most comprehensive data for any given state in the US that we are aware of.

Related Datasets	Time Period	Spatial Regions	Type of Interventions	Data Collection Process	Data Validation
^8^	August 2020 - January 2021	114 Regions, 7 European Countries	17 Types	Participants: Nine researchers, local epidemiologists were consulted Data Sources: administrative divisions	Yes
^9^	May 2020 - July 2020	5,568 municipalities and the Federal District, Brazil	6 Types	Participants: administration officials Data Sources: phone-based survey (47 questions)	No
^6^	January, 2020 - July, 2020	10,129 records, 137 countries	4 Types	Participants: Crowdsourcing Data Sources: Primary: official sources, Secondary: publicly available sources	Yes
^7^	December 2019 - July 2020	56 Countries	8 themes, 63 categories, 500 subcategories	Participants: Crowdsourcing Data Sources: public and government sources	Yes
^13^	January 2020 - March 2021	180 countries and sub-national jurisdictions	19 Types	Participants: Trained Contributors Data Sources: publicly available sources	Yes
^11^	January 2020 - March 2021	186 countries	>10 types	Participants: Authors and Trained Contributors Data Sources: publicly available sources	?
^25^	March - July 2020	1320 US counties	7 Types	Participants: Crowdsourcing Data Sources: publicly available sources	?
^10^	August 2020 - January 2021	114 regions, 7 European Countries	17 Types	Participants: two authors Data Sources: interviews, public resources	Yes
^12^	March 2020 - May 2020	195 countries	16 Types	Participants: Trained RAs, partnered with ML Software Company Data Sources: public resources	Yes
^14^	January 2020 - October 2020	228 countries	13 Types	Participants: RAs Data Sources: ACAPS and UNESCO Datasets	Yes
^21^	March 2020 - April 2020	24 Counties, California	5 types	Participants: Crowdsourcing Data Sources: Surveys	Yes
Our Work	March 2020 - March 2022	120 Counties, Virginia	6 Types	Participants: Trained Contributors Data Sources: publicly available sources	Yes

The dataset presented here provides county-level NPI mandates for the U.S. state of Virginia. The NPI measures are categorized into 6 groups, detailed below, and have been sourced from government sources as well as from social media outlets. Our collection team was composed of a small team of trained undergraduate students. In parallel to the collection process, we performed a systematic data validation for both correctness and completeness. Towards the end of our effort, healthdata.gov published a dataset^26^ containing various state and county policy orders. However, only state-level orders were included for Virginia, while ours is at the county level.

Looking forward, there are several directions where this work can continue: (*i*) expanding this effort to other states in the U.S., (*ii*) continuing the effort to capture nuance within the data - expanding the metadata tag assignment and library of tags, (*iii*) extend analyses of results to further measure the impact of mandates on the course of the pandemic (such as peak analysis and mitigation, the NPI measure impact on mobility, etc.).

Using this dataset, we enable various analyses: the behavior of the local administrations and how they varied by location and through time, the relationship between the counties’ actions as well as with the state mandates, and the impact of NPI measures on COVID-19 cases and hospitalizations. It is important to note that collecting this data will become progressively harder as various sources of information are either discontinued or the knowledge is lost.

## Methods

### Region selection

When determining the scope of the dataset, we chose to focus on a single U.S. state. We chose the U.S. since the team are all native to the U.S. and best understands the local mechanics of information spread. We chose a single U.S. state due to several factors: first, it neutralized issues relating to varying state-level responses to the pandemic (state mandates would be the same for all counties collected), it would lead to a dataset that contained locations relatively close geographically (enabling analyses including mobility between counties), and choosing Virginia specifically meant the team would collect mandates for many counties (Virginia has 3rd most such regions) that had a range of features (politically, topographically, urban/rural, industrial/agricultural, etc.).

### NPI Selection

The NPI data that we collected for each county were the start and end dates of the following interventions: (*i*) closing local businesses, referred to as business_close (b), (*ii*) closing K-12 schools, referred to as school_close (s), (*iii*) closing colleges and universities, referred to as college_close (c), (*iv*) closing places of religious worship, referred to as religion_close (r), (*v*) lockdowns, or broad closure of public and private institutions or asking citizens to stay at home, referred to as lockdown (l) and (*vi*) Mandate to wear masks, referred to as mask_mandate (m). Interventions such as mask mandates usually have associated levels of compliance; we were not able to collect data for this directly. Nevertheless, CMU-Facebook and other surveys have provided a good estimate for this kind of data and can be combined with our dataset in the future. Similarly, we did not collect data on pharmaceutical interventions; this data is available from various official sources and is granular and detailed. It can be combined with NPIs in the future as well. Categorizing mandates into 6 groups ignores a vast array of nuances between mandates of the same type. This was particularly observable with K-12 school closures where a NPI closure might mean that a single elementary school was closed for the period, or the entire school system was closed, for example. In order to proceed with a manageable set of categories, however, we chose these 6. We believe these broadly cover the NPI measures taken by localities that had a material impact on the behavior of their populations.

### Data collection

We had some prior experience with crowdsourced and Amazon Mechanic Turk (AMT) methods^21^. Through this work, the initial methods were promising; we were one of the first groups to start such a campaign. We were able to collect data for a number of states. But in time, the approach faced a several challenges including: (*i*) lack of a standardized workflow, (*ii*) task complexity, (*iii*) long-standing and changing interventions. Together these concerns made it challenging to continue with that approach as the pandemic evolved. As a result, we chose to train a small team to collect the NPI data.

**Fig. 1:**
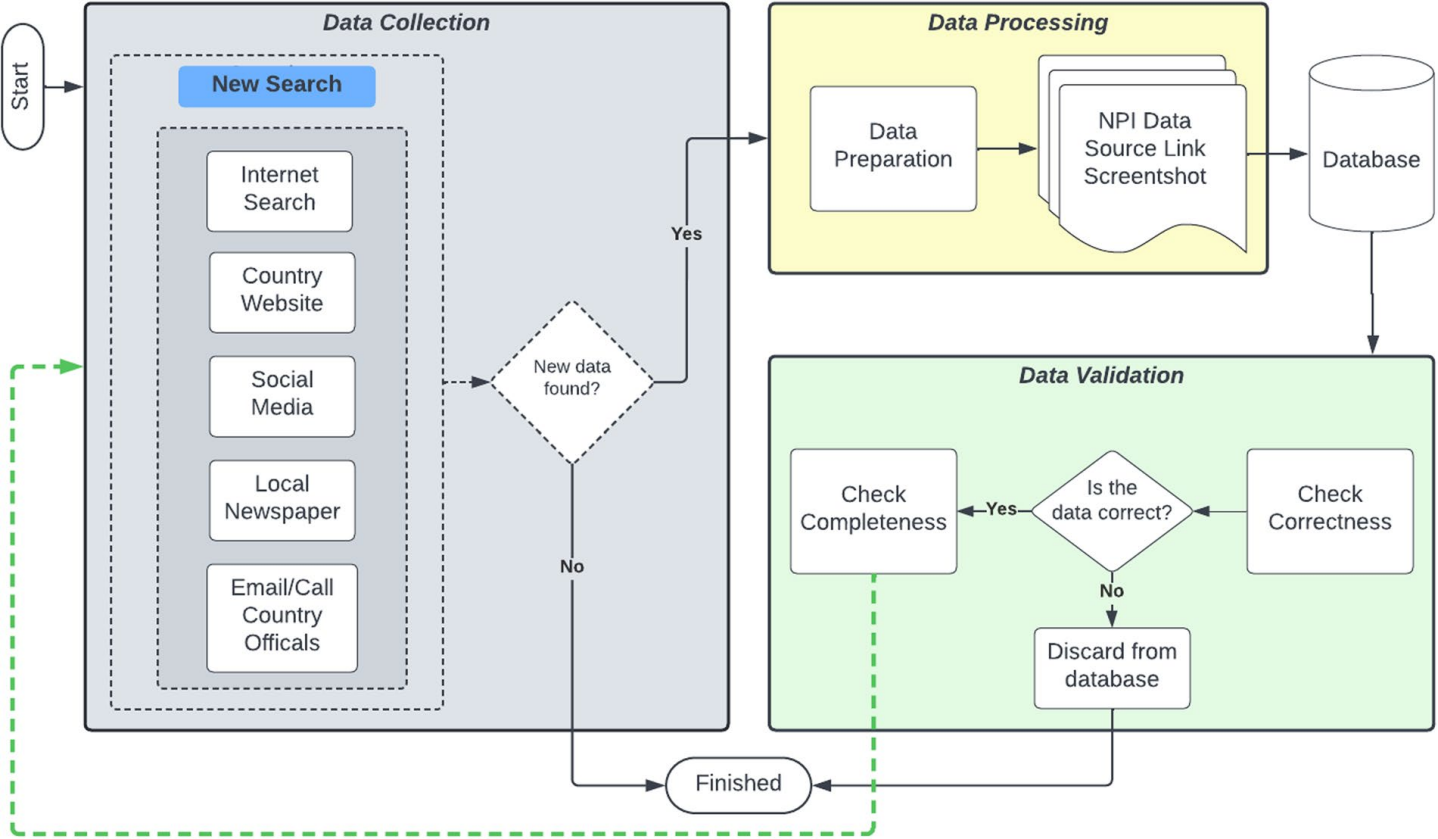
Combined algorithm taken by the collection team. This includes both the data collection and processing phase, and the validation phase. Both begin by selecting a new county.

**Fig. 2:**
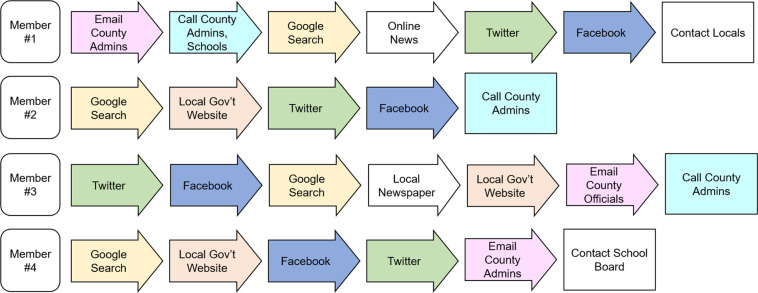
Four examples of the different steps that team members took to find a particular mandate. The individual processes of each team member is different because there was no specific order required by the project. If a mandate was not found by the last step, the member would move on to the next county. The colors in each process indicate a particular location for finding a mandate (Facebook, Twitter, etc.).

**Fig. 3:**
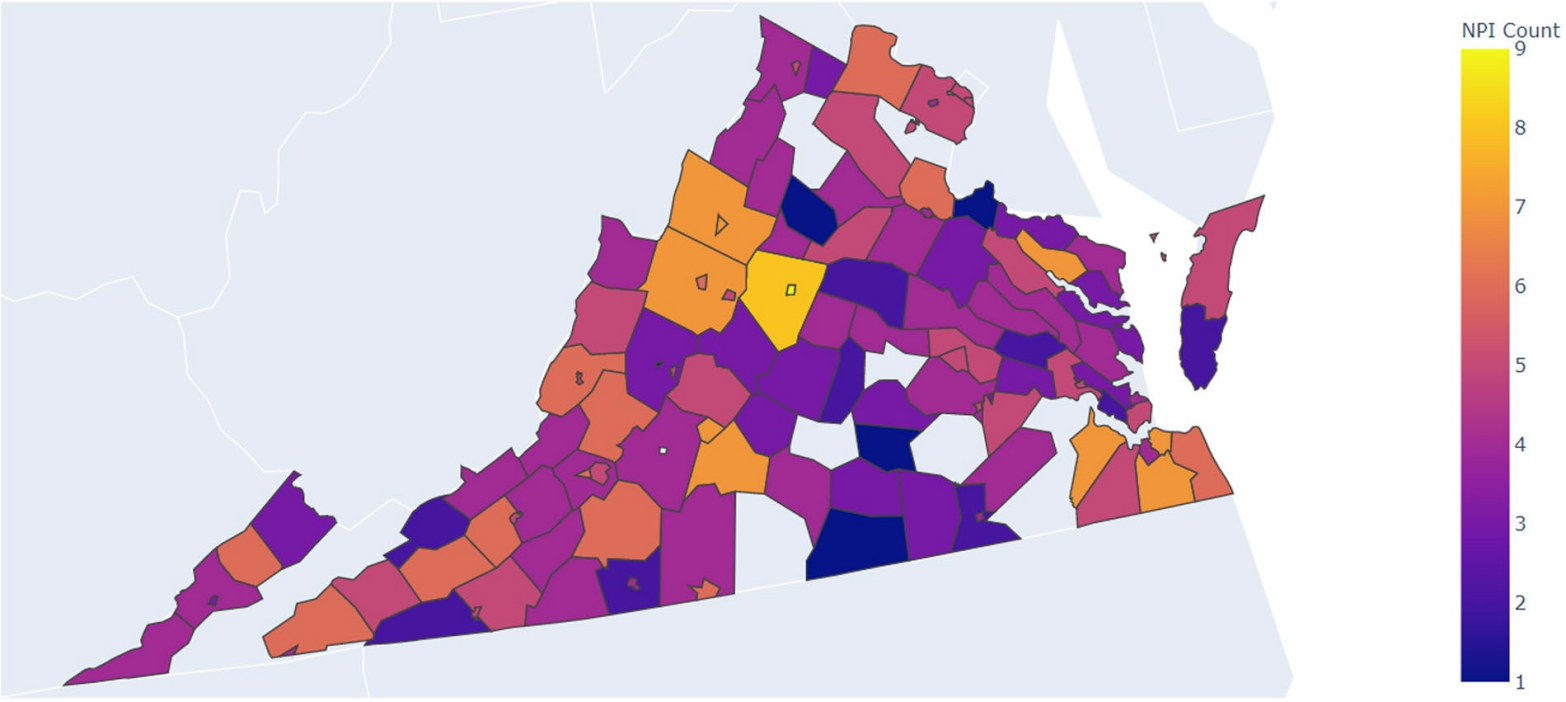
A map showing the total number of NPI mandates found for each county in the state. Blank counties have no known mandates.

**Fig. 4:**
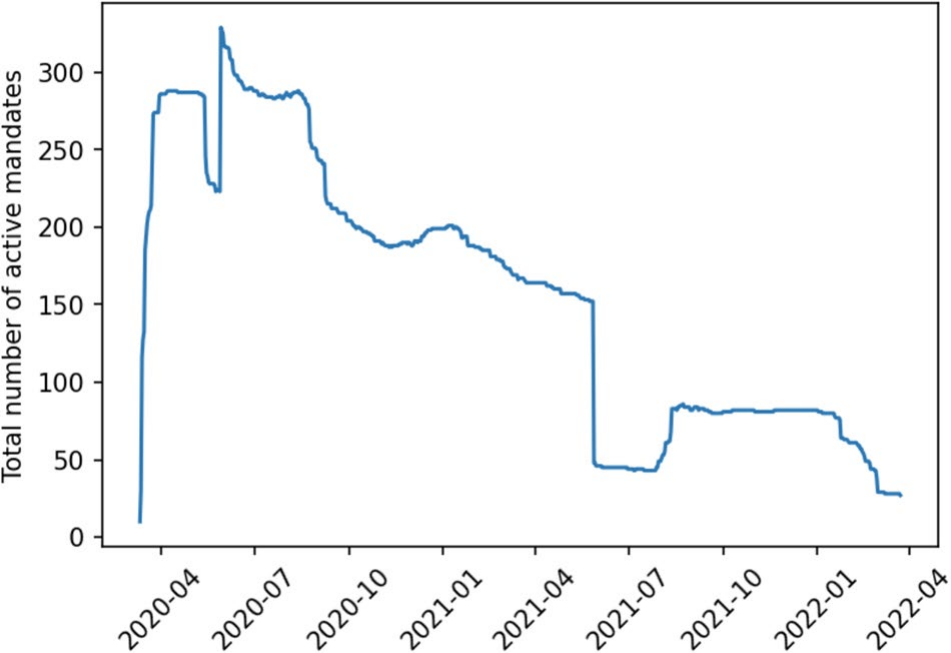
Basic information on the number, duration, and measurement period of the data collected.

**Fig. 5:**
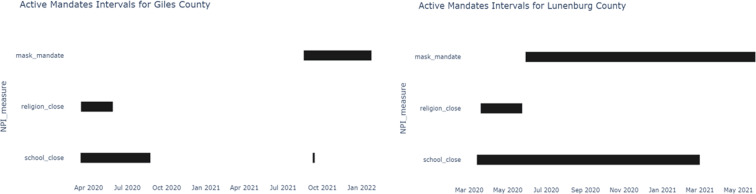
The active periods of the different NPI measures put in place by two counties during the measurement period. The intervals of the mandates (some of which overlap for counties), varies widely between counties.

**Fig. 6:**
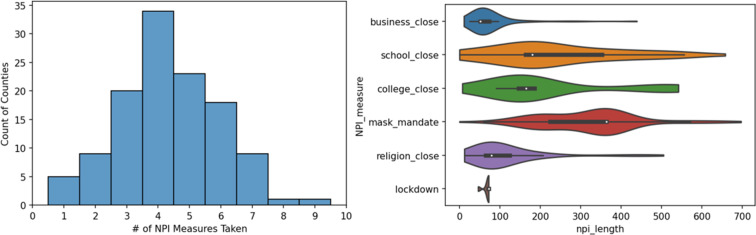
(Left) The distribution of the number of NPI methods taken by counties. Most counties took some NPI measures, few took many. (Right) The distribution of the length of NPI measures. There is a large variation between the different NPI measure lengths. There were few lockdowns communicated by the counties (most following a state-wide stay-at-home order).

**Fig. 7:**
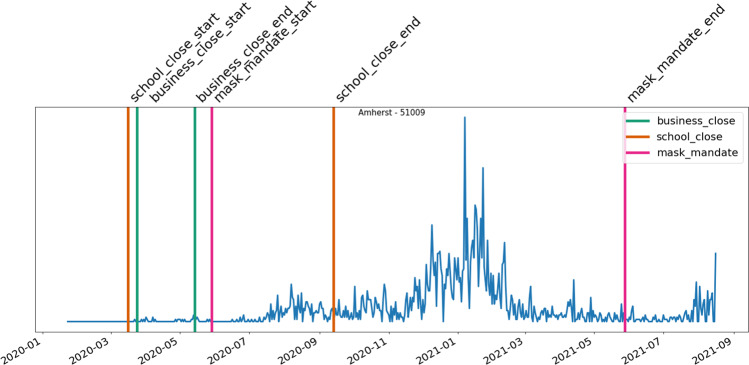
The active periods of the different NPI measures put in place vs. cases in Amherst county during the measurement period.

**Fig. 8:**
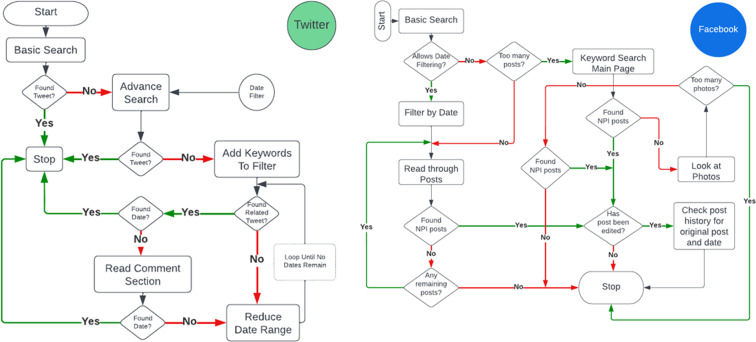
Example of the branching decision process as described by one team member for Twitter (Left) and Facebook (Right).

When we created the global approach to be followed, shown in Fig. 1, we did not give explicit direction to the team members on where or how to find NPI data (the collection sub-process). We gave examples of sources, but we encouraged each to create their own process. This was done for two reasons: first, we believed there was no single most effective way to collect the data. Second, distinct processes between the team members would strengthen the validation steps taken later since each member would approach collecting data for a county differently (and therefore be more likely to find additional information that was not initially found). This resulted in processes that were different on two layers: (*i*) the order of sources, shown in Fig. 2 for different members, and (*ii*) the in-source decision process that each student followed, examples of these processes are shown in Fig. 8.  Fig. 3 shows the total number of NPI mandates found for each county in the state, while Fig. 4 shows a basic view on the number, duration, and measurement period of the data collected. Figs. 5, 6 & 7 have some statistics and county-level visualizations of our data.

Our team was comprised of undergraduate students working closely with faculty. The students were trained on source locations and the individual collection processes were detailed and collected. Due to the nature of the data, the collection process was arduous. It was made difficult by both the scarcity of the NPI communications, the changing availability of NPI information through time, and the complicated decision path required to retrieve the data that existed. As a result, the process was challenging to automate.

When we launched, each of the team members was assigned to collect data for a subset of the Virginia counties. Initially, the counties were split evenly across the team, but the different amounts of available time for the collection eventually meant some team members collected more than others. Regardless of allocation between students, each county was assigned to a single student in order to prevent duplicate entries by different team members for a specific mandate.

### Data post-processing

Once the data was collected, the team followed a standardized process for storing the information. Each data point was recorded in our NPI database (shown in the Data Processing sub-section of Fig. 1). This was a shared database that was added to simultaneously by the members of the team. Since the counties were separated among the team members, there was no risk of overwriting each other’s entries. In addition, the team saved the internet link to the page or source of the mandate (if available), and also took a screenshot of the page or post. This last step was in order to mitigate changes or removals of the data. These images were stored on a shared file repository.

## Data Sources

Our team used several primary sources to collect the information. Since the NPI decisions and communications were produced by local governments, they were published using a range of methods. The team consulted government websites, internet searches, local news organizations, and social media sources (e.g. Facebook, Twitter). In most cases these sources were the administrations’ chosen dissemination channel. In some cases, however, whether due to the passage of time (causing the removal or updating of data, for example) or some other reason, the data would no longer be publicly available. To cover these instances, the team contacted, via email or phone calls, county officials and other county organizations, such as religious groups or county school boards, to acquire the NPI data. Examples of the sources accessed by different team members, and their order, can be seen in Fig. 2.

## Data Records

All collected data has been published in this repository^27^: https://zenodo.org/record/7545486. The latest data has been updated as of June 2022. Our main database contains a .xlsx file (location, mandate type, start date, end date, source links, tags, notes for each date). By the end of the collection period, the data includes mandates for 120 counties (there are 133 counties and independent cities that are county-equivalent in Virginia. For ease of reference, we refer to this entire group as “counties”), or 90% of the state’s total and 509 rows. These counties with data make up 91% of the total population of Virginia. As part of the collection process, we included state-mandated NPI measures (usually a mask mandate) at the county level, since there are a few instances where a county actively chose to not implement the state-mandated NPI measure. Additional information is shown in Table 2.

**Table 2:** Total number of active NPI mandates over time (this includes state mandates at the county level - so a single state mandate is counted for each county that followed it.

Total Mandates	209
Average Mandate Length	226.38 days
Period Start	March 9, 2020
Period End	March 23, 2022
Period Length	745 days
Total Mandates Validated	509
% Mandates Validated	100%

### Main dataset

**FIPS:** FIPS of the county.

**Location name:** Name of the county.

**State name:** Name of the State.

**NPI measure:** Type of NPI measure.

**Start date:** Date the NPI was first started.

**End date:** Date the NPI was first lifted.

**Start link:** Source link of the start date.

**End link:** Source link of the end date.

**Start notes:** Contains tags that apply to both dates, and the start date individually. Tag description can be found in Table 4. This also includes notes on nuances not included in the tags.

**End notes:** Contains tags that only apply to the end date. Tag description can be found in Table 4. This also includes notes on nuances not included in the tags.

**Validated (Correctness):** Binary field indicating if the record was validated for correctness.

**Added during validation (Completeness):** Binary field indicating if the record was added during the validation process.

**Changed start:** Binary field indicating if the start date was changed during validation.

**Changed end:** Binary field indicating if the end date was changed during validation.

## Technical Validation

To ensure the quality of our data, we conducted a two-step validation process: first for correctness, second for completion. To validate for correctness, the team members selected records that they did not produce and checked the saved link and the saved screenshot. If the source data, either the link or the screenshot, matched the record, the record was considered correct and noted in the database. If the link was dead, or the source no longer contained the information (as many websites would update based on the latest communication) and the screenshot absent, the entry was considered decayed and not able to be validated. If the record was incorrect, the team member would update the record (in the event of a typo), or remove the record altogether (in the event of an erroneous source). If the record was a duplicate, it was removed. For the errors in the data, which were generally few, the two most common issues were: incorrect data being entered (whether from misreading or mistyping) and correct NPI data for a different county with the same name (in a different U.S. state).

To validate for completeness, the team member performing the validation would perform their own search for NPI measures for that county, using their own method. Doing this, we were able to try our best to confirm we had captured the accessible information by verifying the database’s completeness for that county. Due to the aforementioned issues of finding the information in the first place, and the trend of removing communications of mandates as time progressed, certainty regarding the completeness of the database is not possible.

The results of our validation is shown in Table 3. The error rate, as a percent, is calculated as (|*m*_*v*_–*m*_*c*_|/(*m*_*v*_))*100 where *m*_*v*_ is the number of mandates validated, and *m*_*c*_ is the subset of validated mandates found to be correct. In Table 2, we show the number and percent of mandates that have been validated at least once. By performing this validation, we have endeavored to ensure the validity of the data that is provided.

**Table 3:** Validation information.

Validation Metric	Correctness	Completeness
Errors Detected	94	2
Error Rate	18.46%	0.4%

**Table 4:** Description of the tags.

Tag ID	Description
Announced	The earliest date the policy was announced
First Date	The first date the policy went into effect
Library	If mandate was about public libraries
Business	Mainly for masks and business, If mandate was about businesses
Northam	If the order was put under Governor Ralph Northam
Youngkin	If the order was put under Governor Glenn Youngkin
State Order	If order put in place by state instead of county or local officials
EO	If executive Order (Also tagged with State Order, so repeat)
Case Increase	If reason given for mandate was increase in cases
All Virtual	All activities virtual
Optional or Option	Option between In Person or Virtual
Hybrid	Hybrid Schedule
In Person	In person option available for some group (not same as hybrid)
Phases	If plan for the opening is in phases (Tagged with “Hybrid” or “In Person”, so repeat)
PreK or 1 or 2 etc	Grades mandate affects
Service	For religious gatherings, if the mandate was about service or mass
Ordinance	If ordinance put into place by county
Local Emergency	If policy is government declaring local emergency
Decided	The date mandate decided (not always the same as “Announced”)
School	Mainly for masks, but if mandate was about schools
Government Employees	Mainly for masks, but if mandate about government employees

Alongside the validation, the team also identified and added some metadata tags to increase the capture of nuance within the mandates. These tags included, for example, whether a mandate was a state-level mandate that was being followed at the county level. Using standardized tags helped maintain a database that could be processed for machine and data analysis.

### Limitations

There are a couple limitations that we have identified in both the approach and the data itself. The replicability of the method is fairly straightforward and does not have material limitations. The resulting process designs from the team members will be different, as expected from a design that allows each member to design their own process. The replicability of the results, however, is severely limited by the changing nature of the underlying data. Using the exact same method (visiting the same sites, using the same searches, etc.) of an individual team member may not yield the same results - a material portion of the data may be no longer accessible, or have moved to a different location.

Regarding the data, the major issue is the decay of information. Source links died, information gets moved or deleted - the information isn’t accessible or no longer exists. This results in many cases of decay where the initial data point is not possible to validate. In addition, the collection activities took place over a series of months from the fall of 2021 through the spring of 2022. To the extent that there were mandates put in place after a team member completed their search for that county, these records may not be included (although some may have been found during the validation phase). Finally, there are likely records that are findable that simply eluded the team members’ searches. The validation process for completeness would not correct the first of these, but should mitigate the second two.

Another limitation regarding the use of the data is the lack of visibility into the compliance with the mandates. Efforts to determine a mandate’s effectiveness will be impacted by the community’s level of compliance. This must be considered for these efforts and additional data should be sought to inform compliance levels, where possible.

Despite these limitations, the data is still useful due to its difficulty to collect and its decaying nature. The data has been validated for correctness and enables analyses to understand the impact of different NPI measures (and combinations thereof) in slowing pandemic spread across counties and the state as a whole. These kind of analyses are important for future decision making.

## Usage Notes

The data enables researchers to analyze and deliver insights into the temporal and spatial relationship between the NPI measures and the counties that enacted them. With analyses such as these, it is possible to inform future decision makers to take a course of action that is supported by historical NPI data and will result in more effective measures to protect (and convince) local populations. This data can be combined with demographic and population information that capture variation between different counties. These additional data, such as economic, mobility, or political features, would broaden the NPI analyses and allow a researcher to attempt to quantify their impact. With these additions, there are plethora of potential uses with two groups provided below.

First, at the state level, researchers can consider three vital questions: (i) which intervention measures had the most impact - which measures should be immediately taken, (ii) what was the impact of the active number of interventions - answering if number of interventions, which is at least a partial proxy for the willingness of an administration to fight the pandemic (i.e. administrations with more and longer NPI measures were more determined to limit the spread of the pandemic), had an impact on its spread, and (iii) were there particular counties whose policies had an out-sized impact on the course of the pandemic for the overall state.

Second, at the county level: (i) the effect of the number of active interventions on pandemic statistics, such as case numbers or hospitalizations, and (ii) which combination of mandate types was most effective for each county. Initially, these two analyses can provide an insight into which counties should take which types of measures during a future pandemic. It may be determined that counties with different demographic and geographic attributes respond to certain NPI measures differently (e.g. mobility constraints may be impactful on largely urban counties). In addition, these can help to understand if a pattern exists in the combination of mandate types or if any combination has done a better job for a cluster of counties.

It is important to note that this data, and the method used to acquire it (barring data decay), can be reused and combined with other similar data sets (either from US counties, for US-based analysis, or other administrations internationally) to build an even more potent data set. As future pandemics, and their likely associated NPI reactions, arise this data will remain reusable to further hone the ideal response.

## Data Availability

No custom code was used to generate the dataset.
